# An analecta of visualizations for foodborne illness trends and seasonality

**DOI:** 10.1038/s41597-020-00677-x

**Published:** 2020-10-13

**Authors:** Ryan B. Simpson, Bingjie Zhou, Tania M. Alarcon Falconi, Elena N. Naumova

**Affiliations:** Tufts University Friedman School of Nutrition Science and Policy, Boston, USA

**Keywords:** Databases, Infectious diseases, Epidemiology, Disease prevention, Research management

## Abstract

Disease surveillance systems worldwide face increasing pressure to maintain and distribute data in usable formats supplemented with effective visualizations to enable actionable policy and programming responses. Annual reports and interactive portals provide access to surveillance data and visualizations depicting temporal trends and seasonal patterns of diseases. Analyses and visuals are typically limited to reporting the annual time series and the month with the highest number of cases per year. Yet, detecting potential disease outbreaks and supporting public health interventions requires detailed spatiotemporal comparisons to characterize spatiotemporal patterns of illness across diseases and locations. The Centers for Disease Control and Prevention’s (CDC) FoodNet Fast provides population-based foodborne-disease surveillance records and visualizations for select counties across the US. We offer suggestions on how current FoodNet Fast data organization and visual analytics can be improved to facilitate data interpretation, decision-making, and communication of features related to trend and seasonality. The resulting compilation, or analecta, of 436 visualizations of records and codes are openly available online.

## Introduction

Disease surveillance systems worldwide face increasing pressure to maintain and distribute data in usable formats with clearly communicated visualizations to promote actionable policy and programming responses^1^. Decade-long efforts to sustain surveillance systems improve early outbreak detection, infection containment, and mobilization of health resources^1–4^ and create adaptive, near-time forecasts for disease outbreaks^5,6^. Web-based platforms provide access to more accurate, timely, and frequent surveillance data. The World Health Organization’s (WHO) FluNet, for example, provides time-referenced data on worldwide influenza^7^. Publicly available downloads increase the flexibility for analyses and enables adaptive research due to frequent and timely reporting.

The pandemic of 2019 novel coronavirus disease (COVID-19) serves as a vivid demonstration of how limited access to publicly available high-quality data can stymy research. As the quantity and diversity of data available for processing, synthesizing, and communicating increases, new visual analytics, including complex multi-panel plots, must be considered to monitor trends, investigate seasonality, and support public health planning^8^. These visualizations, and the methodologies used to generate them, must be standardized to enable comparability across time periods, locations, at-risk populations, and pathogens. However, current surveillance systems, including foodborne disease surveillance in the United States, often compress time series records to simplistic annual trends^9–13^ and describe seasonality by the month(s) with the highest cases per year or the first month of outbreak onset^14–19^. Visualizations using these annual trends or broad assessments of seasonality fail to utilize the full complexity of surveillance data and in some cases may be misleading. More specifically, these visualizations fail to provide detailed examination of how long-term trends change over time, how seasonality estimates vary by year or across locations, or how peak timing and amplitude estimates could change over time.

The CDC Foodborne Disease Active Surveillance Network (FoodNet) provides preprocessed population-based foodborne-disease surveillance records and visualizations via FoodNet Fast, a publicly available data portal^20,21^. The FoodNet Fast platform contains rich demographic data, including age group, gender, and ethnic group, valuable for a broad spectrum of analyses. The visualizations aim to aid users in identifying trends of nine laboratory-confirmed foodborne diseases in select counties from ten US states and nationally. However, in the present form and due to substantial data compression, the available data and visualizations provided are limited in scope preventing the characterization of inter-annual trends and seasonality characteristics and comparisons across locations, diseases, and time^14,15,17,22–24^. The data portal allows researchers to download time series from a public domain. After downloading, processing, and analyzing the data, we created an analecta of visualization techniques enabling inter-annual and sub-annual comparisons between diseases and locations with respect to long-term trends and seasonal oscillations.

Analects, or analecta, is defined as a compilation of select passages from the work of an author^25^. Our analecta includes two compilations: (1) a collection of FoodNet Fast datasets collated into usable and analyzable time series data, and (2) an anthology of effective and informative visualizations for communicating information on disease trends and seasonality. By merging FoodNet Fast datasets and calculating disease rates from counts, we facilitate the comparison of spatial and temporal patterns of foodborne diseases, and detection of potential outbreaks and their synchronization. The presented anthology eases data interpretation, decision-making, and communication of features related to trend and seasonality and can be replicated for other longitudinal disease surveillance databases to support public health interventions.

In this paper, we describe the process of compiling, organizing, and visualizing surveillance time series data using FoodNet Fast as an example^21^. We demonstrate how current FoodNet Fast visual analytics can be improved to provide a clear yet comprehensive description of trends and seasonality features. We emphasize the utility of multi-panel graphs^8^ for describing trends over time and features of seasonality, including disease peak timing and amplitude. We also provide guides on how to explore and compare trends and seasonality between multiple diseases and geographic locations. As surveillance systems offer a time-referenced data repository, analects of data visualizations can show how diseases change over time. Such a tool can help ensure the longevity of data and information to better understand and evaluate the efficacy of public health programming over time. The resulting analecta of 436 visualizations for foodborne diseases, original and processed data, and the codes used to produce visualizations are available at our website (https://sites.tufts.edu/naumovalabs/analecta/).

## Methods

### FoodNet fast data compilation

The FoodNet Fast platform provides publicly available data for laboratory-confirmed diseases caused by seven bacteria [*Campylobacter*, *Listeria*, *Salmonella*, *Shigella*, Shiga toxin-producing *Escherichia coli* O157 and non-O157 (STEC), *Vibrio*, and *Yersinia enterocolitica*] and two protozoa (*Cryptosporidium* and *Cyclospora*) in counties from 10 states: California (CA), Connecticut (CT), Georgia (GA), Minnesota (MN), and Oregon (OR) since 1996; Maryland (MD) and New York (NY) since 1998; Tennessee (TN) since 2000; Colorado (CO) since 2001; and New Mexico (NM) since 2004. National estimates are generated from the sum of data across these 10 states, which represent approximately 15% of the US population^20,21^.

FoodNet Fast allows data download and visualization of these diseases for a user-specified time period. Data downloads include information on the incidence of confirmed cases, monthly percentage of confirmed cases, distribution of cases by pathogen, and totals of cases, hospitalizations, and deaths. For multi-year periods, the portal aggregates totals and monthly percentages into single statistics for the full time period selected rather than showing individual years. This aggregation ensures case anonymity but monthly time units minimize the refinement of trend and seasonality analyses. To calculate monthly percentages of confirmed cases for all diseases in one year and one location, we had to download each state-year combination individually, for a total of 221 files in MS Excel format.

To create a time series of total monthly cases by pathogen and location, we used data from two tables in each data download: annual counts of confirmed cases (long format) and monthly percentage of confirmed cases (wide format). We transposed the monthly percentages of confirmed cases from wide to long format and then multiplied them by the annual counts of confirmed cases (Supplementary Figure S1). Since the provided monthly percentages are rounded to 1 digit in the data download, calculated counts slightly under- or over-estimate annual totals. We did not round non-integer cases in our calculated time series to best preserve the monthly distribution of cases from the original data download. A monthly time series of confirmed cases of hospitalizations or deaths could not be reconstructed as described because no information is provided on their monthly percentages. We next calculated disease rates using confirmed monthly cases and annual population data. Rates are preferred over counts since changes in counts could be a direct result of changes in the population catchment area of a surveillance system. The number of counties and states monitored in FoodNet increased between 1996 and 2004 and has remained constant to date since (Supplementary Table S1).

We downloaded county-level population estimates from the 1990, 2000, and 2010 US Census Bureau interannual census reports, which provide annual population estimates^26–28^. We then estimated state-level FoodNet population catchment area by adding all mid-year (July 1^st^) populations of surveyed counties monitored in each year. Next, we calculated the United States population catchment area by adding all state-level estimates for all surveyed counties for each year. Finally, we developed a time series of monthly rates per 1,000,000 persons for each pathogen and location by dividing monthly counts by annual population estimates and multiplying this quotient by 1,000,000. In addition to monthly rates, we calculated yearly rates by adding all monthly counts each year, dividing by the annual population, and multiplying this quotient by 1,000,000.

### Modeling trends and seasonality

We estimated trend and seasonality characteristics using Negative Binomial Harmonic Regression (NBHR) models, which are commonly used to analyse count-based time series records with periodic fluctuations^29–31^. These models include harmonic terms representing sine and cosine functions, which allow us to fit periodic oscillations. The regression parameters for these harmonic terms serve as a base for estimating important characteristics of seasonality: when the maximum rate occurs (peak timing) and the magnitude at that peak (amplitude). We calculated peak timing, amplitude, and their confidence intervals from NBHR model coefficients using the δ-method, which allow us to transform the regression coefficients of the model to seasonality characteristics based on the properties of the basic trigonometric functions (Supplementary Table S2)^29,30^. To estimate annualized seasonality characteristics, we applied a NBHR model for each study year and location with the length of the time series set to 12 to represent the months of the year. We also estimated seasonality characteristics for the full time period. To show average trends across the entire 22-year period, we fit a NBHR model with three trend terms (linear, quadratic, and cubic) where the length of the time series varied according to when FoodNet began surveying that location from 168 to 264 months. The selection of three polynomial terms was driven by the clarity of interpretation as a monthly increase and the potential for overall acceleration or deceleration, although other ways of assessing the trend such moving averages and spline functions could be also explored.

### Plot terminology

We develop multi-panel visualization techniques using the best practices of current data visualization resources^32,33^ and our own research^8,34,35^. A multi-panel plot, as defined by our earlier work, “involves the strategic positioning of two or more graphs sharing at least one common axis on a single canvas^8^.” These plots can effectively illustrate multiple dimensions of information including different time units (e.g. yearly, monthly), disease statistics (e.g. pathogens, rates, counts), seasonality characteristics (e.g. peak timing, amplitude), and locations (e.g. state-level, national). We use the following common, standardized terminology across visualizations to ensure comprehension:

- Disease – each of the nine reported FoodNet infections, including campylobacteriosis (Camp), listeriosis (List), salmonellosis (Salm), shigellosis (Shig), infection due to Shiga toxin-producing *Escherichia coli* O157 and non-O157 (Ecol), vibriosis (Vibr), infection due to *Yersinia enterocolitica* (Yers), cryptosporidiosis (Cryp) and cyclosporiasis (Cycl)
- Monthly Rate – monthly confirmed cases per 1,000,000 persons
- Yearly Rate – total confirmed cases in a year divided by the mid-year population of all surveyed counties in that location (cases per 1,000,000 persons)
- Frequency – the number of months reporting the disease rates in the same range
- Peak Timing – the time of year according to the Gregorian calendar that a disease reaches its maximal rate; for monthly time series, peak timing ranges in [1, 13[, i.e. from 1.0 (beginning of January) to 12.9 (end of December)
- Amplitude – the mathematical amplitude, or the midpoint of relative intensity; for NBHR models, the amplitude estimate reflects the ratio between the disease rate at the peak (maximum rate) and the disease rate at the midpoint (median rate)
- FoodNet Surveyed County – the counties under FoodNet surveillance as of 2017
- Non-Surveyed County – all remaining counties within a surveillance state as of 2017.

We present our analecta of visualizations allowing to describe trend, examine seasonal signatures, curves depicting characteristic variations in disease incidence over the course of one year, and understand features of seasonality, such as peak timing and amplitude across locations and diseases. We illustrate all visualizations using salmonellosis for the United States from 1996–2017. The full analecta with time series data and code are available on our website (https://sites.tufts.edu/naumovalabs/analecta/) with data and code also available on figshare^36^.

## Results

### Describing trend

The interpretability of trends in a time series plot is greatly affected by the length and units of the time series. FoodNet Fast aggregates data annually, as shown in Supplementary Figure S2, which provides clear, concise information on annual rates. In this example, the rate of salmonellosis remains largely unchanging over time with distinct outbreaks seen in 1999 and 2010. As expected, by compressing data to annual rates, Supplementary Figure S2 masks within-year trends of disease rates. FoodNet reports and publications similarly tend to show only inter-annual changes in disease counts or rates^9–13,37,38^. Without more granular within-year variations, the viewer cannot determine if increased yearly rates are driven by erratic outbreaks in a specific month or higher rates across all months of the year.

**Fig. 1:**
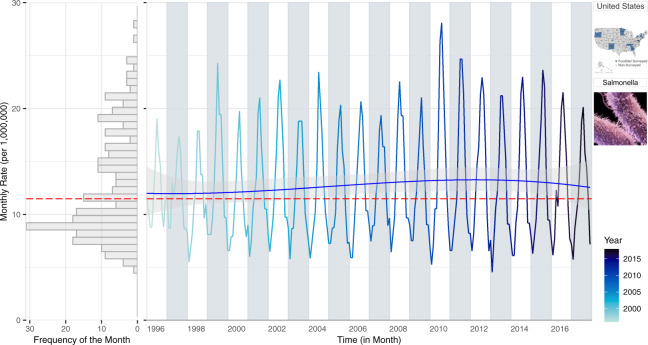
A multi-panel plot: a rotated histogram of monthly rate frequency (left panel) sharing the vertical monthly rate-axis with the time series of monthly rates (right panel) for salmonellosis in the US from 1996–2017. The red line indicates the median rates while the blue line is a NBHR model fit with seasonal oscillators and three (linear, quadratic, and cubic) trend terms.

To capture within-year trends, we propose a multi-panel plot that combines information on monthly rates, inter-annual trends, and the frequency distribution of rates by utilizing the shared axes of individual plots (Fig. 1). The right panel of Fig. 1 provides a time series of monthly rates with a NBHR model fit with three trend terms (linear, quadratic, and cubic). The inclusion of polynomial terms allows us to capture long-term trends (linear term) and their acceleration and deceleration over time (quadratic and cubic terms). The predicted trend line is shown in blue and its 95% confidence interval is in grey shades. The estimated median monthly rate is shown in red. The left panel depicts a rotated histogram of rate frequencies indicating the right-skewness of the monthly rate distribution. The histogram shares the vertical monthly rate-axis with the time series plot and is essential for connecting two concepts: the distribution of monthly counts on the base of their frequency and the distribution of monthly counts over time. Two pictograms refer to the selected pathogen and location.

Figure 1 shows the stability of seasonal oscillation in salmonellosis over time series with increased rates from 1998–2010 followed by a gradual decrease in rates through 2017. While preserving the within-year seasonal fluctuations, the plot provides additional information. Alternating background colours help distinguish differences in the shape of seasonal curves between adjacent years. An increasingly darker hue for the monthly rate values distinguishes more recent data from more historic data. Contrasting background colours mixed with a gradual intensity of line hues, saturation, brightness, and transparency allow for greater focus and attention to trends in the data^32–34^.

The rotated histogram in the left panel of Fig. 1 shows the distribution of monthly rates and its degree of skewness due to months with high counts. We include the red median line to provide the most appropriate measure of central tendency for the skewed distribution. The shared vertical axis helps readers track those high values to a specific month in the time series. The distribution also justifies the use of negative binomial regression models to evaluate temporal patterns. By supplementing the time series plot with the distribution of monthly rates, we show a visual rationale for using appropriate analytical tools (negative binomial model, in this case) for calculating inter-annual trends.

### Examining seasonal signatures

FoodNet reports describe seasonal patterns with the month with the highest cases per year^13^. Supplementary Figure S3 provides an example of a typical seasonal curve from the FoodNet Fast portal. The estimates are the average percentage of confirmed cases per month for salmonellosis in the US from 1996–2017. This visualization provides easily computed and interpreted information on seasonality: on average, salmonellosis cases peak in August for this 22-year period. However, the data compression and use of relative measurements, such as average percentages, masks variability in monthly values across years. Supplementary Figure S3 leaves certain questions unanswered. Do seasonal patterns vary by year? Is this gradual incline and decline in the signature stable over the 22-years or masking frequent erratic outbreaks? What is the variability in cases at each time of year? What are the actual rates for each month of the year for each year?

**Fig. 2:**
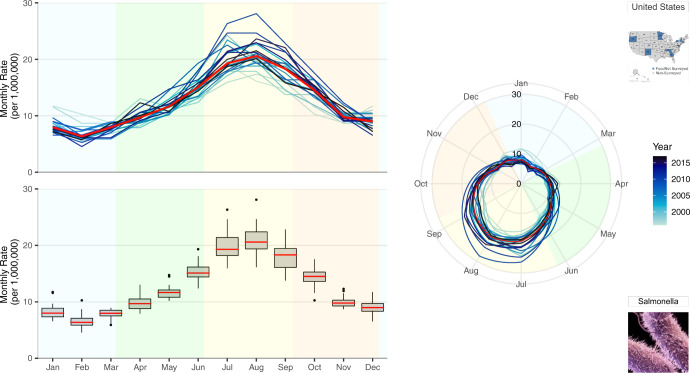
A multi-panel plot for visualizing seasonal signatures of salmonellosis monthly rates in the US from 1996–2017. This includes overlaid annual time series plots of monthly rates, a box plot of average monthly rates for the 22-year period, and overlaid annual radar plots of monthly rates. Background colours indicate the four seasons defined by solar solstices and equinoxes: winter (blue), spring (green), summer (yellow), and autumn (orange).

To better understand annual differences in seasonal behaviors, we propose a multi-panel plot that incorporates annual seasonal signatures, summary statistics of monthly rates, and radar plots (Fig. 2). Given varying visual perceptions of these three ways of presenting seasonal patterns, we offer side-by-side comparisons that aim to increase comprehension. The top-left panel provides an overlay of all annual seasonal signatures, a set of curves depicting characteristic variations in disease incidence over the course of one year, where line hues become increasingly darker with more recent data and a red line indicates median monthly rates, as in Fig. 1. The bottom-left panel provides a set of box plots for each month that aggregates information over the study period and provides essential summary statistics, including the median rate values and the measures of spread. The shared horizontal axis allows the two plots to be compared across the years using identical scales. To provide visual context, background colours were used to indicate the four seasons (winter, spring, summer and autumn). The right panel provides overlaying monthly rates using a radar plot where time is indicated on the rotational axis and rates are indicated on the radial axis. The radar plot emphasizes the periodic nature of seasonal variations in one continuous line with graduating colours. The colour hue of the lines, background colour, median line colour and the axis scales are uniform across all three panels. We also repeat the pictograms to refer to the selected pathogen and location.

For salmonellosis, disease rates are highest in the summertime (with peaks in July and August) and lowest during the wintertime (with a well-defined February nadir). Rate increases and decreases during equinox periods indicate bacterial growth rates due to more and less favourable climate conditions, respectively.

The top-left panel of Fig. 2 disaggregates annual seasonal signatures to show the stable seasonal peak timing of salmonellosis across all years. This stable behavior reflects the average monthly percentages shown in Supplementary Figure S3. This panel provides the seasonal signatures of salmonellosis rates and helps to better understand the annual variations hidden in Supplementary Figure S3. Supplementary Figures S4 and S5 provide examples of how occasional irregular outbreak behaviors could be identified using this annual overlay plotting technique.

The bottom-left panel of Fig. 2 overcomes another deficiency of Supplementary Figure S3 of masking variations. This panel provides box plots depicting the median monthly rate (red), interquartile range (box), 95% confidence interval (whisker), and outliers or potentially influential observations (markers) over the 22-year period. Measures of distribution spread provide an insight for the dispersion of rates in each month: the variability of salmonellosis rates decreases in winter months closer to the February nadir but increases in summer months of July and August closer to the seasonal peak. Unusually high values are indicative of erratic behavior characterized by spikes in specific months and years.

The right-hand panel of Fig. 2 further emphasizes the periodic nature and the positioning of the seasonal peaks and nadirs. Radar or spider plots describe time using a rotational axis where the radial distance from the centre of the plot depicts rate magnitude^39–42^. Radial axes, compared to perpendicular axes, show annual fluctuations as a continuous flow. This more clearly demonstrates declines of salmonellosis rates during nadir months (November to March) without the visual discontinuity of left panel visuals.

**Fig. 3:**
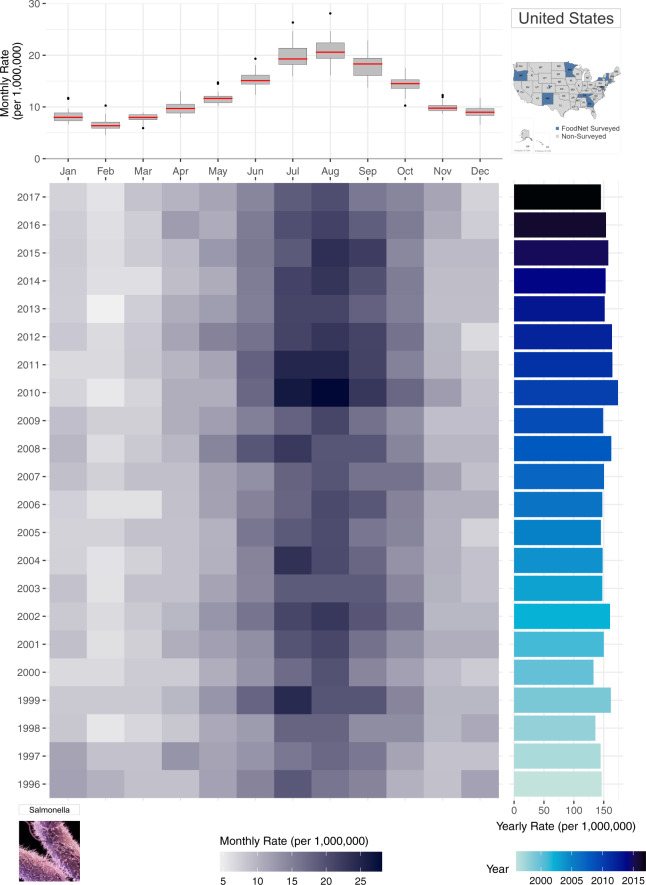
A multi-panel plot for improved visualization of the annual seasonal signatures of monthly rates of salmonellosis in the US from 1996–2017. The top panel provides a box plot of monthly rates for each month of year. The bottom heatmap shows the distribution of monthly rates for each year where darker hues indicate greater rates. The right panel provides a rotated bar graph of yearly rates.

To capture the advantage of a multi-panel plot (Fig. 3), we incorporate the boxplot from Fig. 2 (lower left panel) with a calendar heatmap containing 264 monthly rate values. In the heatmap, information for each individual year is shown as stacked rows of width 12 (for each month of the year) where cell colour intensity represents the magnitude of monthly rates. Like Fig. 2, the heatmap illustrates the highest rates (shown as the darker cells) are in July and August. Compared to stacked line plots, however, Fig. 3 provides an individual row for each year of the time series, allowing for greater decomposition, differentiation, and comparison of seasonal signatures across years. In this plot, seasonal changes are shown horizontally from left to right - from January to December and the yearly trend transition can be observed in a vertical view from bottom to top-from year 1996 to 2017 in the right panel.

While Fig. 2 provides the annual variability of seasonal patterns, monthly rate values for each year are difficult to ascertain. Instead, the emphasis is placed on similarities and differences of the seasonal curvature over time. In Fig. 3, the attention shifts to comparing the intensity of rates per month of the year across years. Here, we evaluate which months of the year are most intense across years using the intensity of each cell’s colour hue to describe the intensity of rates. The Fig. 3 panel integrates information on both trends and seasonality along with the individual monthly values unlike any of the previously shown visualizations. Yearly rates provide a bar graph for comparing fluctuations in inter-annual rates while the adjacent heatmap indicates the month(s) driving these fluctuations. In doing so, the calendar heatmap identifies whether inter-annual changes are driven by sporadic outbreaks or increased seasonal magnitude of rates. At the same time, the shared axis box plot provides an overview of the average seasonal signature for the entire time series, as emphasized in Fig. 2.

### Understanding seasonal features

**Fig. 4:**
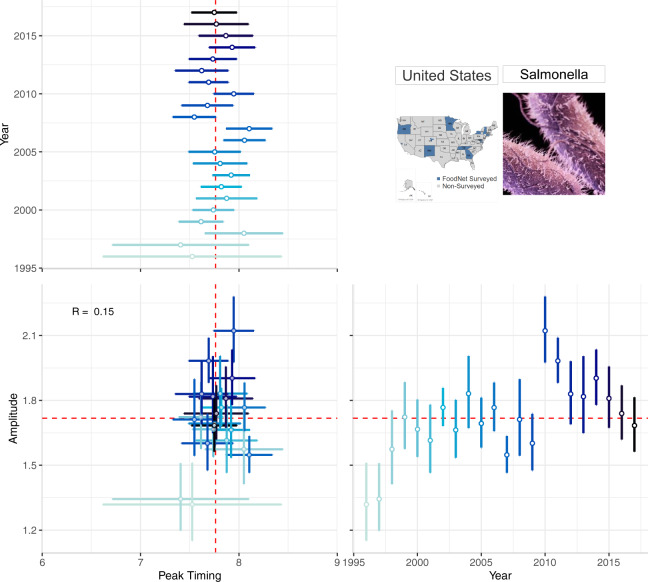
A multi-panel plot for visualizing the annual peak timing and amplitude of salmonellosis in the US from 1996–2017. The top-left panel shows the peak timing of salmonellosis by year; the bottom-right panel shows the annual amplitude by year. The bottom-left panel shows their combination: a scatterplot between peak timing and amplitude. Marker colour intensity indicates more historic vs. more recent data, horizontal and vertical whiskers provide measures of uncertainty, and red lines indicate median peak timing and amplitude across 22 years.

Detailed characterization of the timing and intensity of seasonal peaks requires a standardized estimation of peak timing and amplitude. This standardization improves upon implemented techniques of comparing months with the highest cases in a given year by applying the δ–methods to NBHR model parameters^29,30^. Average seasonality characteristics can be estimated across the full time series while annual estimates allow for more granular comparisons between years. To depict point estimates and confidence intervals of seasonality characteristics, we use forest plots - a technique commonly used in meta-analyses^18,43,44^. We develop a multi-panel forest plot to depict annual peak timing, annual amplitude, and their joint distribution, to better understand the relationship among the seasonal features and how it changes over time (Fig. 4).

Figure 4 is a multi-panel plot that incorporates two forest plots (one each for annual peak timing and amplitude estimates) and one scatterplot (for peak timing and amplitude) to describe seasonality features. The top-left panel shows peak timing estimates (as month of the year, ranging from 1.0 (beginning of January) to 12.9 (end of December) - horizontal axis) for each study year (vertical axis). The bottom-right panel shows amplitude estimates where the horizontal axis indicates the study year and the vertical axis shows the amplitude (ratio between the disease rate at peak and the median rate). The bottom-left corner shows the scatterplot of peak timing (horizontal axis) and amplitude (vertical axis) with markers representing each pair of annual estimates. Measures of uncertainty (95% confidence intervals) are reflected in error bars of each marker; dashed red lines show median peak timing and amplitude estimates.

Forest plots in Fig. 4 provide a compact, clear, and comprehensive visual describing the stability of peak timing and amplitude, even without showing the entire seasonal signature. For example, salmonellosis peak timing and amplitude vary little each year indicating strong, stable seasonal peaks in July and August. Consistent peak timing means practitioners could time preventive strategies, increase awareness for foodborne illnesses to prevent transmission, and inform food retailers of when food safety inspections should be in higher demand within their supply chains. Consistent amplitude estimates show that the intensity of salmonellosis varies little over time, suggesting that federal food safety regulations have not greatly influenced the number of salmonellosis cases annually. This type of information is likely to benefit FoodNet Fast users.

Supplementary Figure S6 provides an example of how a sporadic outbreak behavior can be depicted by forest plots of peak timing and amplitude estimates for shigellosis in NY. The lack of seasonality for shigellosis is shown by the broad confidence intervals for peak timing, spanning the entire year and beyond.

### Drawing comparisons across locations

**Fig. 5:**
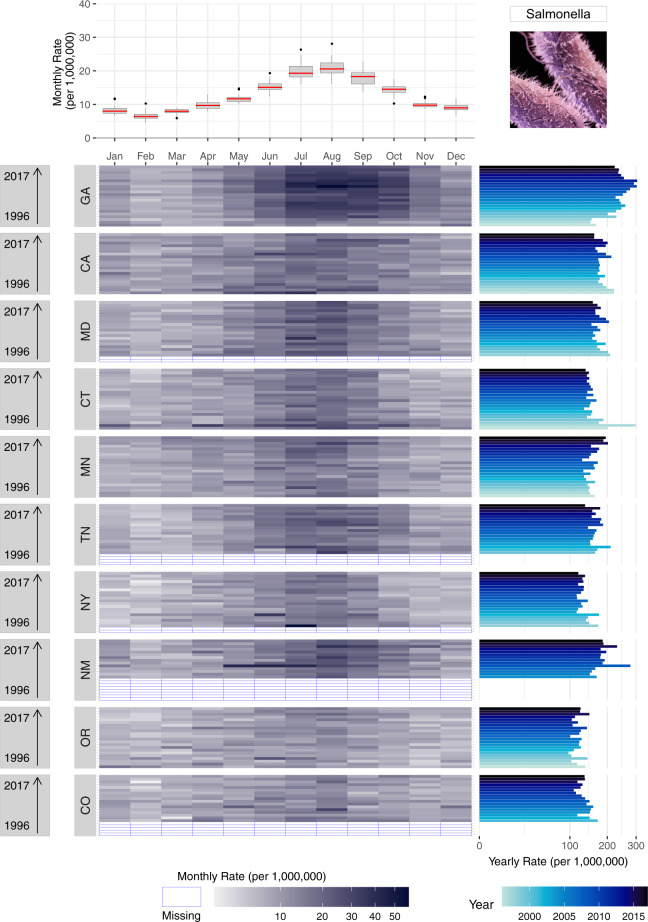
A multi-panel plot for comparing seasonal signatures and yearly rates of salmonellosis in the US from 1996–2017 disaggregated by the ten FoodNet-surveyed states. The top panel provides a box plot of monthly rates for each month of year for the US. The calendar heatmap uses shared horizontal axes to show the distribution of monthly rates for each year and each state. Darker hues indicate higher rates while empty cells with blue borders indicate years when FoodNet surveillance was not conducted. The right panel provides a rotated bar graph of yearly rates. Given sizable differences in rates across states we applied a high-order calibration colour scheme.

**Fig. 6:**
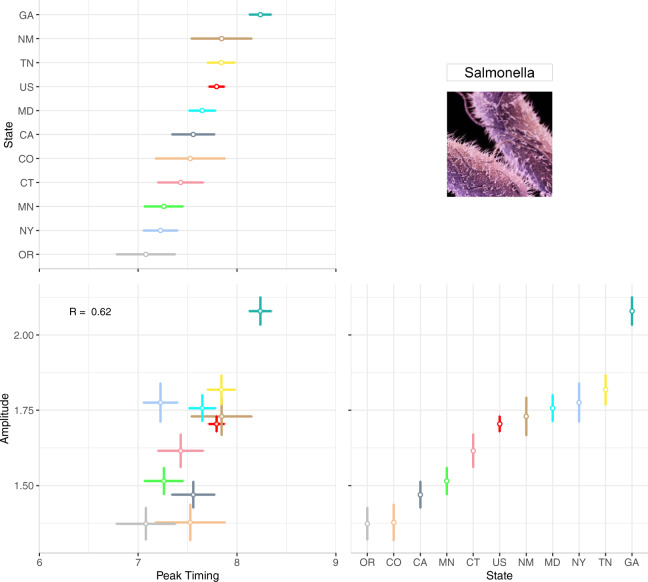
A multi-panel plot for visualizing the annual peak timing and amplitude of salmonellosis in ten FoodNet-reporting states and the US from 1996–2017. The top-left panel shows the average peak timing of salmonellosis per location while the bottom-right panel shows the average amplitude per location. The bottom-left panel shows a combined scatterplot between peak timing and amplitude estimates.

Supplementary Figure S7 provides an example bar chart showing differences in the average annual incidence of salmonellosis for the ten FoodNet-surveyed states. As with other FoodNet visualizations, data has been compressed to show only average annual estimates. Like in Fig. 1, annual rates mask within-year seasonal variations, calling into question if differences in states are driven by single year outbreaks. The alphabetical organization of the horizontal axis makes states ranking and comparison more difficult than if they were ordered from highest to lowest rates. To ease comparisons of a single disease across geographic locations, we generated two multi-panel plots (Figs. 5 and 6). These plots mirror the same techniques shown above but include multiple shared axes and multiple locations to draw spatial comparisons.

Supplementary Figure S8 follows the same design as Fig. 3; we replicate this design for salmonellosis in all FoodNet-surveyed states. We present all states in one plot in a descending order by the sum of yearly rates in each state and display all available data so that state level patterns can be compared. The box plot in the top panel provides an overview of the seasonal signature for the entire US. The bottom panel disaggregates the entire US by states. As shown, all states share similar peak timing in July and August for almost every surveillance year from 1996–2017. For some states, like GA and CA, rates are densely concentrated from July to September with rapid decline from September to February and gradual incline from February to July. For other states, like NY and OR, seasonal peaks are much less pronounced and rate differences are smaller between months. Clear indication of missing data provides additional information on differences in reporting completeness not captured by previous figures.

While heatmaps provide information on seasonal signatures, yearly rate bar graphs (right panel) capture state-level trends over time. States are stacked in the order of total cases from 1996–2017, showing differences in the intensity of salmonellosis infection across states. Comparisons within states between years help identify inter-annual rate changes over time. For example, while MD and CA have generally declined in annual rates over the 22-year period, GA rates increased from 1996–2012 and steadily declined from 2012–2017. In combination with heatmaps, yearly rates also allow for detailed assessment of sporadic outbreaks. For example, erratic outbreaks came from two monthly spikes in April and June for CT in 1997 while for NM in 2000 a multi-month outbreak lasted from May to July.

By using shared horizontal and vertical axes, this plot eases the comparison of disease rates across months, years and states. It also helps to determine hotspots and detect potential co-occurrences of infection in different states. Moreover, the plot can be periodically updated by adding new information, offering a sustainable approach to make consistent comparisons between historical data and data captured in the future.

To compare seasonality features across locations, we designed a multi-panel plot similar to Fig. 4 to show average peak timing and amplitude estimates over the 22-year period for each state. In Fig. 6 the top-left panel plots peak timing estimates ordered from the earliest (OR) to latest (GA) peak timing while the bottom-right panel plots amplitude estimates in order of magnitude. Marker and line colours are used to differentiate the seasonality feature estimate and its measure of uncertainty between states. The bottom-left panel shows the relationship between peak timing and amplitude across states.

### Comparisons across diseases

**Fig. 7:**
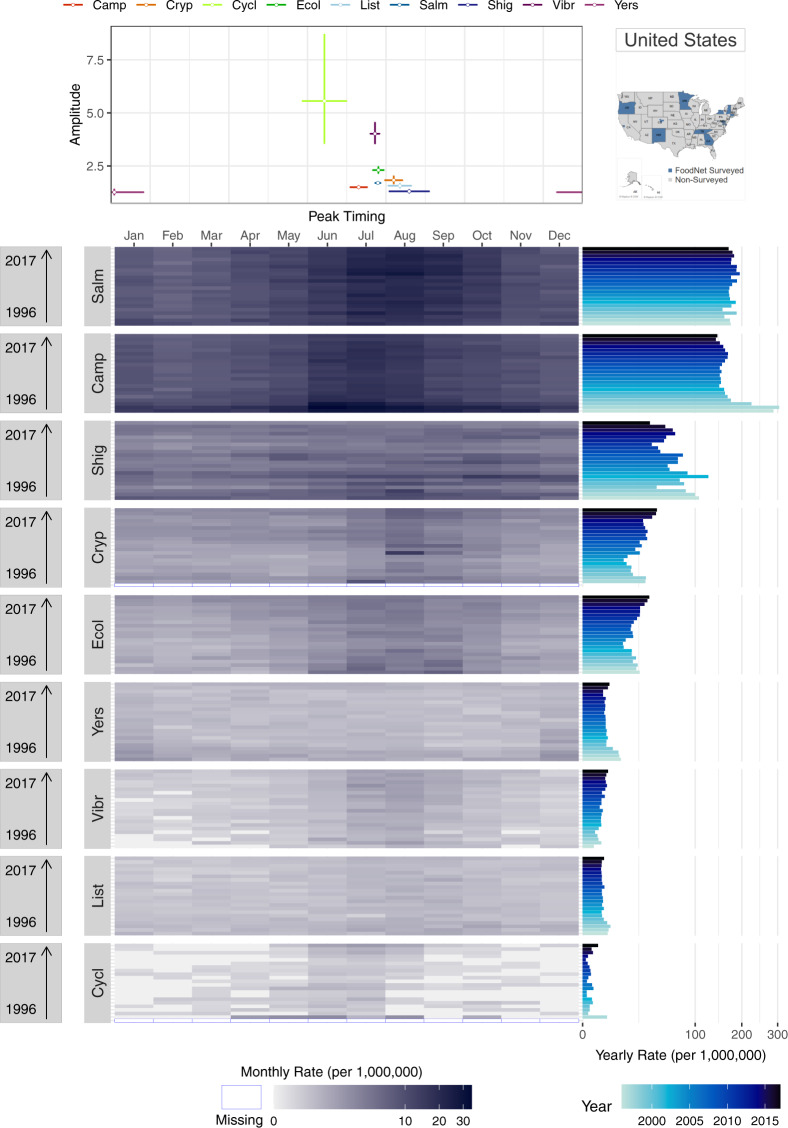
A multi-panel plot for comparing seasonal signatures and yearly rates of nine FoodNet-reported infections in the US from 1996–2017. The top panel provides a scatterplot of average peak timing and amplitude estimates per pathogen across the 22-year period. The bottom heatmap uses shared horizontal axes to show the distribution of monthly rates for each year and each disease (campylobacteriosis (Camp), listeriosis (List), salmonellosis (Salm), shigellosis (Shig), infections caused by Shiga toxin-producing *Escherichia coli* O157 and non-O157 (Ecol), vibriosis (Vibr), infections caused by *Yersinia enterocolitica* (Yers), cryptosporidiosis (Cryp) and cyclosporiasis (Cycl)). Darker hues indicate greater rates while empty cells with blue borders indicate years when FoodNet surveillance was not conducted. The right panel provides a rotated bar graph of yearly rates.

**Fig. 8:**
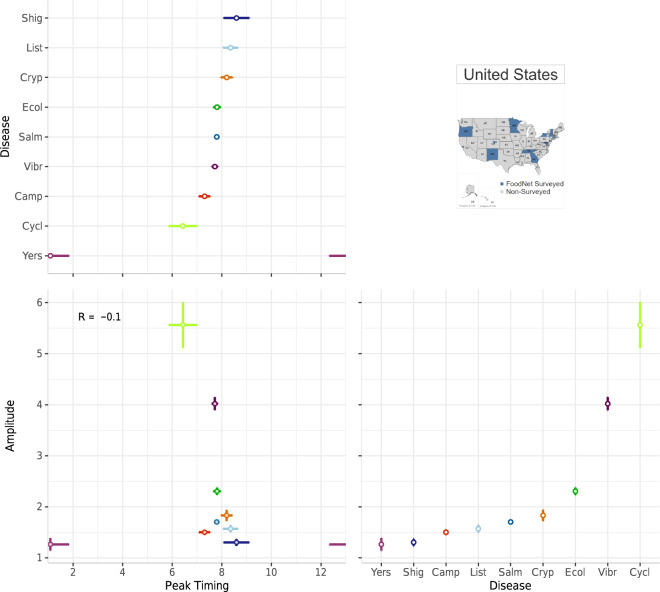
A multi-panel plot for visualizing the average peak timing and amplitude of nine FoodNet-reported pathogens in the US from 1996–2017. The top-left panel shows the average peak timing of each disease while the bottom-right panel shows the average amplitude per disease (campylobacteriosis (Camp), listeriosis (List), salmonellosis (Salm), shigellosis (Shig), infections caused by Shiga toxin-producing *Escherichia coli* O157 and non-O157 (Ecol), vibriosis (Vibr), infections caused by *Yersinia enterocolitica* (Yers), cryptosporidiosis (Cryp) and cyclosporiasis (Cycl)). The bottom-left panel shows a combined scatterplot between peak timing and amplitude estimates.

Supplementary Figure S8 provides an example of FoodNet Fast bar chart showing differences in the total confirmed infections for each of the nine surveyed pathogens in the US from 1996–2017. The visual shows that infections due to *Campylobacter* and *Salmonella* have the highest cumulative counts of infections while *Cyclospora* has the lowest counts. While depicting these differences clearly, this visual lacks sufficient specificity for drawing more intricate comparisons between infections. How are counts or rates distributed by year? What are the within-year variations of rates by pathogen? How do seasonal signatures and their variability differ by pathogen? Can axes be reordered or recalculated for easier comparisons between pathogen counts or rates? We propose two multi-panel plots (Figs. 7 and 8) that improve the comparisons of multiple diseases for a given geographic location.

Figure 7 replicates the plot design of Fig. 5 but emphasizes comparisons between pathogens for a single location. Instead of a seasonal signature box plot, the top panel provides a scatterplot to illustrate the peak timing and amplitude of each pathogen. In combination with the heatmap in the bottom panel, these plots illustrate the strong seasonality of salmonellosis, campylobacteriosis, and STEC in July and August and cryptosporidiosis in August. These seasonal peaks are consistent across almost all years suggesting a stable seasonal periodicity and strong alignment between infections. In contrast, infections caused by *Yersinia enterocolitica*, vibriosis, listeriosis, and cyclosporiasis have much less pronounced seasonality and monthly rates much lower than salmonellosis or campylobacteriosis. Yearly rates, shown in the right panel, indicate erratic outbreak behaviors for cyclosporiasis. Given sizable differences in rates across diseases we applied a high-order calibration colour scheme.

We also provide the same multi-panel, shared-axis visualization design seen in Fig. 6 for comparisons across pathogens. Figure 8 includes a forest plot of peak timing by disease pathogen (top-left panel), a forest plot of amplitude by pathogen (bottom-right panel), and a scatterplot between peak timing and amplitude estimates (bottom-left panel). As in Fig. 6, average peak timing and amplitude estimates are calculated using NBHR models for the entire 22-year time series. Comparisons between diseases allow for understanding the alignment of seasonal processes across pathogens as well as shared relative magnitudes in a specific location. In our case, most of the pathogens peak during the summertime except cyclosporiasis. However, if the selected diseases peak during winter months, we recommend adjusting the starting and ending months to center these peaks in the figure.

## Discussion

In this study we offered ways of thinking on how public data platforms can be improved by using visual analytics to provide a comprehensive description of trends and seasonality features in reported infectious diseases. We emphasize the utility of multi-panel graphs by showing side-by-side different methods of depicting trends over time and features of seasonality, including disease peak timing and amplitude. We provided visual tools to show trends (Fig. 1), examine seasonal signatures (Figs. 2 and 3) and their characteristics (Fig. 4), compare diseases across locations for trends (Fig. 5) and seasonal signatures (Fig. 6), and drawing comparisons across pathogens for trends (Fig. 7) and seasonal signatures (Fig. 8). We also provide guides on how to explore and compare trends and seasonality between multiple diseases and geographic locations using FoodNet Fast data. Given varying visual perceptions, we offer side-by-side comparison of different tools aiming to increase comprehension and faster adoption of efficient graphical depictions.

We developed a time series of monthly rates by reconstructing a time series of monthly counts (see Fig. 1) then dividing counts by the sum of all FoodNet-surveyed counties’ mid-year populations per state per 1,000,000 persons. In this calculation, we recognize that average monthly percentages are rounded in the raw data file and do not sum to 100% annually for downloaded years. This rounding resulted in obtaining non-integer counts within our time series. To prevent modification of raw data files, we did not round counts to integers before or after calculating rates. No information is provided on the FoodNet Fast website for the definition of confirmed cases, and data downloads provide no metadata for distinguishing cases from hospitalizations and deaths. Although the case definition is provided on the CDC website as “laboratory-confirmed cases (defined as isolation for bacteria or identification for parasites of an organism from a clinical specimen) and cases diagnosed using culture-independent methods”^45^, it forces the user to assume that a confirmed case is any person with laboratory confirmed cultures of a specific pathogen who may or may not have been hospitalized or died from infection. FoodNet also collects information on hospitalizations and deaths, but does not provide information on the monthly percentage of hospitalizations or deaths, so users are unable to reconstruct a monthly time series for deaths or hospitalizations.

The FoodNet Fast platform states all confirmed diseases as “incidence” calculations. Technical documentation on the FoodNet website shows that the term incidence reflects cases per 100,000 persons (used interchangeably with a disease rate) with no distinction of whether these are newly introduced within the population (i.e. incidence) or the total persons diagnosed with a disease (i.e. prevalence)^46^. We found that monthly rates can similarly be calculated by multiplying annual incidence rates and the monthly percentage of confirmed cases for each disease-state pair. Differences between our calculations and this alternative method are no more than ± 2%. We suspect that rounding errors of average monthly percentages and differential population catchment areas for rate calculations cause these differences. As shown in Supplemental Table S1, population of the surveillance catchment area is changing over time. Oftentimes, publicly available surveillance datasets, including FoodNet Fast, do not include location- and year-specific population catchment area estimates, which are needed for calculating rates from diseases counts. As FoodNet does not provide population catchment areas for calculating rates, it forces the user to assume that FoodNet surveillance reaches the total population of a surveyed county (likely an overestimate), yet such oversight is easy to fix. Three collaborators confirmed our monthly rate calculations for quality control.

We applied the negative binomial harmonic regression NBHR models, commonly used in the time series analysis of counts and cases. While the use of NBHR models, specifically the inclusion of trigonometric harmonic oscillations, is similar to existing works on foodborne illnesses, these studies often incorporate harmonic oscillators only to adjust for or remove seasonal oscillations^47–55^. We have extended the use of harmonic terms and develop the tools to estimate peak timing and amplitude^8,29,30^. The developed δ-method provides a systematic calculation of confidence intervals for peak timing and amplitude estimates based on the results of harmonic regression models. In the proposed approach, we present the amplitude as the ratio of seasonal peak to seasonal median, which offers robust estimation even for rare or highly sporadic infections. These features are not available when traditional models, like Auto-Regressive Integrated Moving Average (ARIMA), are applied^56^. Measures of uncertainty enable formal testing and comparisons across diseases in the same location or locations for the same disease. In our previous works, we have demonstrated the broad utility of the δ-method and applications of peak timing and amplitude estimation in the context of epidemiological studies^6,29,56–62^.

We evaluate each state’s cases individually as well as all national cases as the sum of all states’ cases. Our analysis evaluated all cases reported to FoodNet Fast irrespective of demographic factors such as age group, sex, or ethnic group. Future analyses can consider conducting analyses using demographic factors available on the FoodNet Fast platform such as age group (<5, 5–9, 10–19, 20–29, 30–39, 40–49, 50–59, 60–69, 70 + years), sex (male and female), and ethnic group (American Indian and Alaskan Native, Asian and Pacific Islander, Black, Multiple, White). To incorporate this information, our methodology for data extraction would need to be repeated for each subcategory or combination of categories desired (e.g. download 221 files for males and 221 files for females). Future analyses can also consider differences in pathogen strain, which can only be obtained if extracting data for each pathogen-location-year combination (e.g. 221 files for each of the 9 diseases for each of 11 locations or 21,879 files).

FoodNet Fast, like many global disease surveillance databases, has no metadata describing missing data. FoodNet Fast reports missing counts using “N/A” for years when pathogens or locations were not under surveillance. However, there are also years when FoodNet surveillance was live in a state, but a pathogen is missing from the data download. We believe that this missing data comes when, for a given year, a pathogen has 0 total cases. However, we cannot specify whether absences of surveillance reporting came due to a breakdown in reporting or 0 annual counts. Without specification, we have set any year with “N/A” as missing due to no reported case information.

When calculating peak timing and amplitude using the δ-methods, we applied NBHR models adjusted for harmonic seasonal oscillators and three trends (linear, quadratic, and cubic). We selected the polynomial terms as an example, yet researchers can consider alternative techniques for measuring seasonality such as splines, nonparametric regression, ARIMA models, or their extensions. Additionally, the CDC recommends using a mixed effects model when conducting time series analyses on FoodNet Fast data to account for differential population catchment areas and laboratory culture confirmation techniques pre- and post-2004^1,2,46^. We focus on the analysis of individual states and diseases and adjust for population catchment variations by calculating monthly rates using county-level population estimates. Future analyses could include detailed assessments between peak timing and amplitude across diseases, locations, and time periods. Such analyses will help determine whether a synchronization of outbreak peaks occurs or if social, economic, or environmental factors influence peak timing and amplitude.

### Future applications

This analecta of visualizations intends to communicate detailed information on foodborne outbreak trends and seasonality suitable for a general audience, public health professionals, stakeholders, and policymakers. Future applications would involve the development of an interactive web-based platform allowing users to select the outcome, timeframe, and location of interest for educational training and research purposes. For example, public health researchers and practitioners could use this tool to generate insights related to long-term trends, changes in disease dynamics, or changes in populations at risk^62^. Information on when and where outbreaks are most common enable producers, distributors, and retailers to improve food safety practices to prevent these outbreaks. Finally, this platform could aid policymakers in shaping public understanding of outbreak dynamics and using scientific evidence to refine public health policies.

## Supplementary information

Supplementary Information

## Data Availability

The analecta of our time series of monthly rates, 436 data visualizations, and code used for all calculations and visualizations are available on our website (https://sites.tufts.edu/naumovalabs/analecta/). Data and code can be directly downloaded from the website while visualizations are linked on the website to an external visualization repository. Time series data and code are also available on figshare^36^. Visualizations on our website are provided in the same order as presented here: describing trends (Fig. 1), examining seasonal signatures with the three standard techniques: line graphs, boxplots, and radar plots (Fig. 2) and heatmaps (Fig. 3), characterizing features of seasonality (Fig. 4), drawing comparisons across locations for trends (Fig. 5) and seasonal signatures (Fig. 6), and drawing comparisons across pathogens for trends (Fig. 7) and seasonal signatures (Fig. 8). File downloads are available for trend, seasonal signature, and annual time series visualizations. For images examining a single disease in a single location, downloads are formatted where the prefix abbreviates the location and the suffix abbreviates the pathogen (see Supplementary Table S3). For visualizations comparing multiple locations or diseases, the prefix “LOC” indicates comparisons across locations while the prefix “DIS” indicates comparisons across pathogens (see Supplementary Tables S4,S5).

## Code availability

All statistical analyses were conducted using STATA (SE 15.1) software. All visualizations were created using R Version 3.6.2 and Tableau Professional 2019.1 software. All software code is open access on our website (https://sites.tufts.edu/naumovalabs/analecta/) and figshare, and is available for public reuse with proper citation of this manuscript^36^.
