# Supplementary material for: An analecta of visualizations for foodborne illness trends and seasonality
Source: Sci Data. 2020 Oct 13;7:346. doi: 10.1038/s41597-020-00677-x (PMC7553952; doi:10.1038/s41597-020-00677-x)
Supplement: Supplementary file 1 — Supplementary Information [file 41597_2020_677_MOESM1_ESM.docx]

**An analecta of visualizations for foodborne illness trends and seasonality**

### Authors

Ryan B. Simpson^1^, Bingjie Zhou^1^, Tania M. Alarcon Falconi^1^, Elena N. Naumova^1^

**Affiliations**

1. Tufts University Friedman School of Nutrition Science and Policy

corresponding author(s): Elena N. Naumova (elena.naumova@tufts.edu)

### Supplementary Materials

**Supplementary Table S1.** Estimates of the population catchment area (in millions) of the FoodNet surveillance system using Census annual population counts in FoodNet-surveyed counties………….. **3**

**Supplementary Table S2.** Equations for estimating a) trend with linear, quadratic, and cubic trend terms, b) peak timing and amplitude for annualized time series and full time series based on log-linear Negative Binomial Harmonic Regression (NBHR) models [29-30]…………………………………………...……… **4**

**Supplementary Table S3.** An example of the file download table for visualizations describing trend, examining seasonal signatures, and understanding features of seasonality]………………………………….. **5**

**Supplementary Table S4.** An example of the file download table for visualizations comparing trends and seasonal signatures across FoodNet-reported pathogens within each FoodNet-surveyed state and nationally.…………………………………………...….………………………………..…………………………………………...……… **6**

**Supplementary Table S5.** An example of the file download table for visualizations comparing trends and seasonal signatures across FoodNet-surveyed states (and nationally) for each FoodNet-reported pathogen..…………………………………………...….………………………………..…………………………………………...………**7**

Supplementary Figure S1. Steps followed to create a time series of monthly foodborne disease cases. 8

Supplementary Figure S2. FoodNet Fast time series plot of annual rates of foodborne diseases caused by *Salmonella* in the US from 1996-2017. This plot was generated on 05 December 2019 using the FoodNet Fast portal [20] by selecting *Salmonella* as the pathogen. 9

Supplementary Figure S3. A FoodNet Fast line plot showing the percentage of confirmed diseases by month caused by *Salmonella* in the US from 1996-2017. This plot was generated on 05 December 2019 using the FoodNet Fast portal [20] by selecting *Salmonella* as the pathogen of interest. 10

Supplementary Figure S4. A multi-panel plot for visualizing seasonal signatures of cryptosporidiosis monthly rates in the US from 1996-2017. Here, outbreaks in 1997 and 2005 are visible in all three panels compared to the stable seasonal behavior of the disease. Background colours indicate the four seasons defined by solar solstices and equinoxes: winter (blue), spring (green), summer (yellow), and autumn (orange). 11

Supplementary Figure S5. A multi-panel plot for visualizing seasonal signatures of listeriosis monthly rates in the US from 1996-2017. While the box plot in the bottom-left panel depicts a stable seasonal signature, the top-left and right panels show the irregular behavior of each annual seasonal signature. Background colours indicate the four seasons defined by solar solstices and equinoxes: winter (blue), spring (green), summer (yellow), and autumn (orange). 12

Supplementary Figure S6. A multi-panel plot for visualizing the annual peak timing and amplitude of shigellosis in New York (NY) from 1996-2017. The top-left panel shows the peak timing of shigellosis by year; the bottom-right panel shows the annual amplitude by year. The bottom-left panel shows their combination: a scatterplot between peak timing and amplitude. Background colours indicate the four seasons defined by solar solstices and equinoxes: winter (blue), spring (green), summer (yellow), and autumn (orange). Marker colour intensity indicates more historic vs. more recent data, horizontal and vertical whiskers provide measures of uncertainty, and red lines indicate median peak timing and amplitude across 22 years. 13

Supplementary Figure S7. A FoodNet Fast bar chart showing the average annual incidence caused by *Salmonella* across all ten FoodNet-surveyed states. This plot was generated on 05 December 2019 using the FoodNet Fast portal [20] by selecting *Salmonella* as the pathogen of interest. The ¶ symbol indicates states where FoodNet does not survey all counties. 14

Supplementary Figure S8. A FoodNet Fast bar chart showing the number of confirmed infections by pathogen for the US from 1996-2017. This plot was generated on 05 December 2019 using the FoodNet Fast portal [20] by not selecting any state (default is national estimates). 15

**Supplementary Table S1.** Estimates of the population catchment area (in millions) of the FoodNet surveillance system using Census annual population counts in FoodNet-surveyed counties.

| **State** | **County** | **Population in catchment area (in millions) per year** | | | | | | | | |
| --- | --- | --- | --- | --- | --- | --- | --- | --- | --- | --- |
|  |  | **1996** | **1997** | **1998** | **1999** | **2000** | **2001** | **2002** | **2003** | **2004 – 2017** |
| **California** | Alameda and San Francisco | 1.89 | 1.92 | 1.94 | 1.96 | 2.23 | 2.25 | 2.23 | 2.22 | 2.22 – 2.54 |
|  | Contra Costa |  |  |  |  | 0.95 | 0.97 | 0.98 | 0.99 | 0.99 – 1.15 |
| **Colorado** | Adams, Arapahoe, Denver, Douglas, and Jefferson |  |  |  |  |  | 2.10 | 2.15 | 2.19 | 2.21 – 2.76 |
|  | Boulder and Broomfield |  |  |  |  |  |  | 0.31 | 0.32 | 0.32 – 0.39 |
| **Connecticut** | Hartford and New Haven | 1.62 | 1.62 | 1.68 | 1.62 | 1.68 | 1.68 | 1.69 | 1.70 | 1.72 – 1.76 |
|  | Rest of State |  |  | 1.59 | 1.66 | 1.72 | 1.73 | 1.74 | 1.76 | 1.77 – 1.83 |
| **Georgia** | Clayton, Cobb, Dekalb, Douglas, Fulton, Gwinnett, Newton, and Rockdale | 2.72 | 2.81 | 2.88 | 2.91 | 3.16 | 3.21 | 3.25 | 3.28 | 3.32 – 4.10 |
|  | Barrow, Bartow, Carroll, Cherokee, Coweta, Fayette, Forsyth, Henry, Paulding, Pickens, Spalding, and Walton |  | 0.85 | 0.90 | 0.95 | 0.99 | 1.03 | 1.08 | 1.13 | 1.17 – 1.61 |
|  | Rest of State |  |  |  | 3.92 | 4.37 | 4.38 | 4.44 | 4.66 | 4.66 – 4.72 |
| **Maryland** | Anne Arundel, Baltimore, Baltimore City, Carroll, Harford, and Howard |  |  | 2.44 | 2.45 | 2.52 | 2.53 | 2.55 | 2.57 | 2.59 – 2.76 |
|  | Montgomery and Prince George's |  |  |  |  |  | 1.71 | 1.73 | 1.75 | 1.76 – 1.97 |
|  | Rest of State |  |  |  |  |  |  | 1.15 | 1.18 | 1.20 – 1.32 |
| **Minnesota** | All Counties | 4.65 | 4.69 | 4.73 | 4.78 | 4.93 | 4.98 | 5.02 | 5.05 | 5.09 – 5.58 |
| **New Mexico** | All Counties |  |  |  |  |  |  |  |  | 1.90 – 2.09 |
| **New York** | Genesee, Livingston, Monroe, Ontario, Orleans, Wayne, and Yates |  |  | 1.11 | 1.10 | 1.13 | 1.13 | 1.13 | 1.13 | 1.13 – 1.14 |
|  | Albany, Columbia, Greene, Montgomery, Rensselaer, Saratoga, Schenectady, and Schoharie |  |  |  | 0.98 | 0.99 | 0.99 | 1.17 | 1.01 | 1.01 – 1.04 |
|  | Erie, Niagara, and Wyoming |  |  |  |  |  |  | 1.04 | 1.03 | 1.03 – 1.18 |
|  | Allegany, Cattaraugus, Chautauqua, Chemung, Schuyler, Seneca, Steuben, Warren, and Washington |  |  |  |  |  |  |  | 0.81 | 0.64 – 0.64 |
|  | Clinton, Delaware, Essex, Franklin, Fulton, Hamilton, and Otsego |  |  |  |  |  |  |  |  | 0.34 – 0.34 |
| **Oregon** | All Counties | 3.20 | 3.24 | 3.28 | 3.32 | 3.43 | 3.47 | 3.51 | 3.55 | 3.57 – 4.14 |
| **Tennessee** | Cheatham, Davidson, Dickson, Hamilton, Knox, Robertson, Rutherford, Shelby, Sumner, Williamson, and Wilson |  |  |  |  | 2.88 | 2.91 | 2.95 | 2.86 | 2.90 – 3.48 |
|  | Rest of State |  |  |  |  |  |  |  | 2.98 | 3.01 – 3.24 |
| **United States** | All FoodNet Reporting Counties | 14.08 | 15.13 | 20.55 | 25.65 | 30.98 | 35.07 | 38.12 | 42.17 | 44.55 – 49.78 |

**Supplementary Table S2.** Equations for estimating a) trend with linear, quadratic, and cubic trend terms, b) peak timing and amplitude for annualized time series and full time series based on log-linear Negative Binomial Harmonic Regression (NBHR) models [29-30].

| Feature | Equation |
| --- | --- |
| Full NBHR Model, applied to full time series to demonstrate the overall trend | $\ln\left( E\left[ Y_{t} \right] \right)= \beta_{0} + \beta_{1}\left( t \right)+ \beta_{2}\left( t^{2} \right)+ \beta_{3}\left( t^{3} \right)$  where *Y_t_* – time series of monthly rates; $\beta$ reflect the contribution of linear, quadratic, and cubic trend terms. |
| Annualized or Full NBHR Model, applied to 12 consecutive months for a given year, or to a full time series of the study ranging from 168 to 264 months | $ln(E[Y_{t}]) = \beta_{0} + \beta_{s}(sin(2\pi\omega t)) + \beta_{c}(cos(2\pi\omega t))$  where *Y_t_* – time series of monthly rates; sin(2πωt) and cos(2πωt) define seasonal oscillations of frequency ω=1/M, where M=12 to represent the length of the annual cycle in months. |
| Peak Timing (P_T_),  as estimates based on the regression coefficients for two harmonic terms: sin(2πωt) and cos(2πωt) and their 95% confidence intervals (CI) | $\tan\emptyset=\frac{\sin\emptyset}{\cos\emptyset}$, thus: $\emptyset=arctan\left( \frac{\beta_{s}}{\beta_{c}} \right)$  $\mathrm{Var}\left( \emptyset\right)= \frac{{\beta_{c}}^{2}{\sigma_{s}}^{2}+ {\beta_{s}}^{2}{\sigma_{c}}^{2}- {2\sigma}_{\beta_{s}\beta_{c}}\beta_{s}\beta_{c}}{\left( {\beta_{c}}^{2}+ {\beta_{s}}^{2} \right)^{2}}$  if $\beta_{s}>0 \& \beta_{c} >0$, then: $P_{T}= \left( \emptyset\right)\left( \frac{M}{2\pi} \right)$  if $\beta_{c}<0$, then: $P_{T}= \left( \emptyset+\pi\right)\left( \frac{M}{2\pi} \right)$  if $\beta_{s}<0 \& \beta_{c} >0$, then: $P_{T}= \left( \emptyset+2\pi\right)\left( \frac{M}{2\pi} \right)$  $95\% CI \left( P_{T} \right)= P_{T} \pm1.96\sqrt{Var(\emptyset)}\left( \frac{M}{2\pi} \right)$ |
| Amplitude ($\gamma$),  as estimates based on the regression coefficients for two harmonic terms: sin(2πωt) and cos(2πωt) and their 95% confidence intervals (CI) | $\gamma= e^{\vartheta}$, where $\vartheta=\sqrt{{\beta_{c}}^{2}+ {\beta_{s}}^{2}}$  $\mathrm{Var}\left( \vartheta\right)= \frac{{\beta_{c}}^{2}{\sigma_{c}}^{2}+ {\beta_{s}}^{2}{\sigma_{s}}^{2}+ {2\sigma}_{\beta_{s}\beta_{c}}\beta_{s}\beta_{c}}{{\beta_{c}}^{2}+ {\beta_{s}}^{2}}$  $95\% CI \left( \gamma\right)= e^{\vartheta\pm1.96\sqrt{\mathrm{Var}\left( \vartheta\right)}}$ |

**Supplementary Table S3.** An example of the file download table for visualizations describing trend, examining seasonal signatures, and understanding features of seasonality.

|  | **Camp** | **List** | **Salm** | **Shig** | **Ecol** | **Vibr** | **Yers** | **Cryp** | **Cycl** |
| --- | --- | --- | --- | --- | --- | --- | --- | --- | --- |
| **CA** | CA_Camp | CA_List | CA_Salm | CA_Shig | CA_Ecol | CA_Vibr | CA_Yers | CA_Cryp | CA_Cycl |
| **CO** | CO_Camp | CO_List | CO_Salm | CO_Shig | CO_Ecol | CO_Vibr | CO_Yers | CO_Cryp | CO_Cycl |
| **CT** | CT_Camp | CT_List | CT_Salm | CT_Shig | CT_Ecol | CT_Vibr | CT_Yers | CT_Cryp | CT_Cycl |
| **GA** | GA_Camp | GA_List | GA_Salm | GA_Shig | GA_Ecol | GA_Vibr | GA_Yers | GA_Cryp | GA_Cycl |
| **MD** | MD_Camp | MD_List | MD_Salm | MD_Shig | MD_Ecol | MD_Vibr | MD_Yers | MD_Cryp | MD_Cycl |
| **MN** | MN_Camp | MN_List | MN_Salm | MN_Shig | MN_Ecol | MN_Vibr | MN_Yers | MN_Cryp | MN_Cycl |
| **NM** | NM_Camp | NM_List | NM_Salm | NM_Shig | NM_Ecol | NM_Vibr | NM_Yers | NM_Cryp | NM_Cycl |
| **NY** | NY_Camp | NY_List | NY_Salm | NY_Shig | NY_Ecol | NY_Vibr | NY_Yers | NY_Cryp | NY_Cycl |
| **OR** | OR_Camp | OR_List | OR_Salm | OR_Shig | OR_Ecol | OR_Vibr | OR_Yers | OR_Cryp | OR_Cycl |
| **TN** | TN_Camp | TN_List | TN_Salm | TN_Shig | TN_Ecol | TN_Vibr | TN_Yers | TN_Cryp | TN_Cycl |
| **US** | US_Camp | US_List | US_Salm | US_Shig | US_Ecol | US_Vibr | US_Yers | US_Cryp | US_Cycl |

**Supplementary Table S4.** An example of the file download table for visualizations comparing trends and seasonal signatures across FoodNet-reported pathogens within each FoodNet-surveyed state and nationally.

|  | **CA** | **CO** | **CT** | **GA** | **MD** | **MN** | **NM** | **NY** | **OR** | **TN** | **US** |
| --- | --- | --- | --- | --- | --- | --- | --- | --- | --- | --- | --- |
| **Across Diseases** | DIS_CA | DIS_CO | DIS_CT | DIS_GA | DIS_MD | DIS_MN | DIS_NM | DIS_NY | DIS_OR | DIS_TN | DIS_US |

**Supplementary Table S5.** An example of the file download table for visualizations comparing trends and seasonal signatures across FoodNet-surveyed states (and nationally) for each FoodNet-reported pathogen.

|  | **Camp** | **List** | **Salm** | **Shig** | **Ecol** | **Vibr** | **Yers** | **Cryp** | **Cycl** |
| --- | --- | --- | --- | --- | --- | --- | --- | --- | --- |
| **Across Locations** | LOC_Camp | LOC_List | LOC_Salm | LOC_Shig | LOC_Ecol | LOC_Vibr | LOC_Yers | LOC_Cryp | LOC_Cycl |


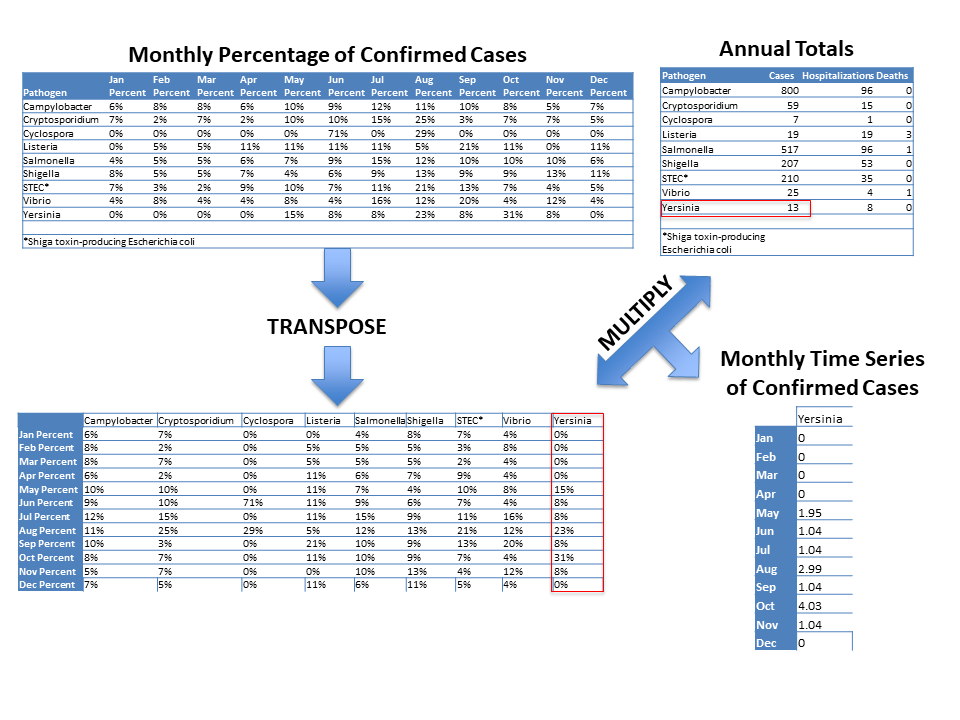


Supplementary Figure S1. Steps followed to create a time series of monthly foodborne disease cases.

**
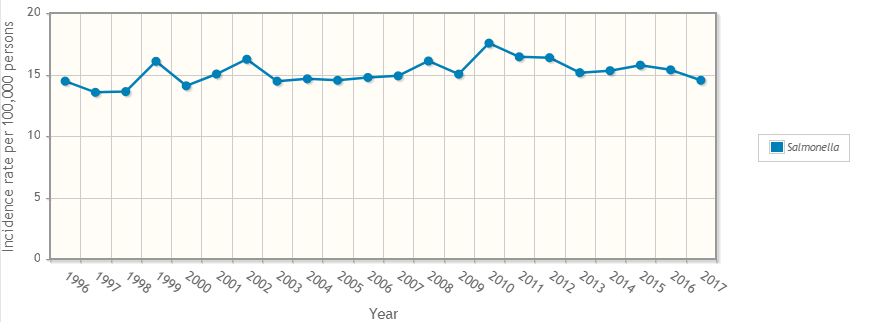
**

Supplementary Figure S2. FoodNet Fast time series plot of annual rates of foodborne diseases caused by *Salmonella* in the US from 1996-2017. This plot was generated on 05 December 2019 using the FoodNet Fast portal [20] by selecting *Salmonella* as the pathogen.


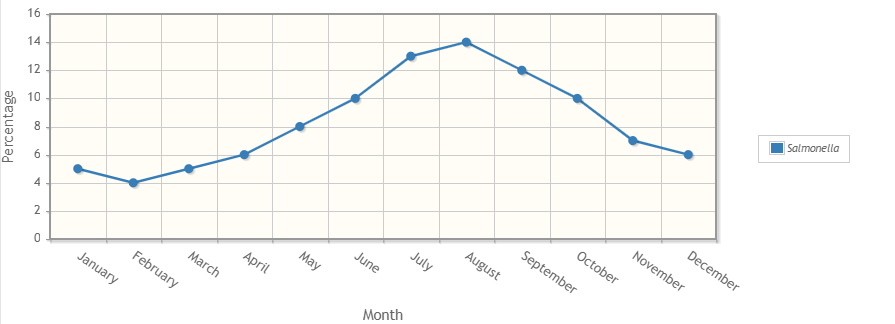


Supplementary Figure S3. A FoodNet Fast line plot showing the percentage of confirmed diseases by month caused by *Salmonella* in the US from 1996-2017. This plot was generated on 05 December 2019 using the FoodNet Fast portal [20] by selecting *Salmonella* as the pathogen of interest.


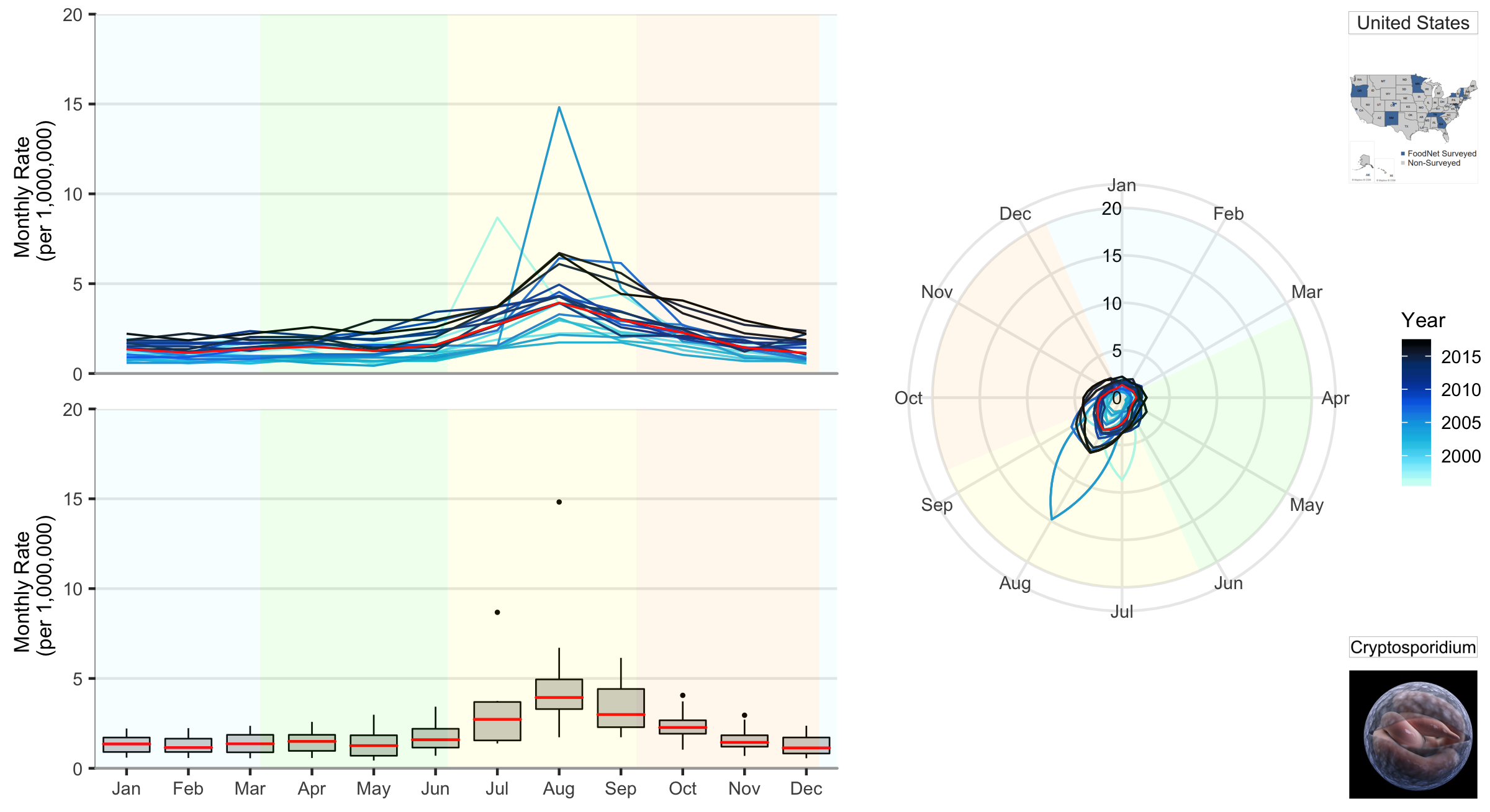


**Supplementary Figure S4.** A multi-panel plot for visualizing seasonal signatures of cryptosporidiosis monthly rates in the US from 1996-2017. Here, outbreaks in 1997 and 2005 are visible in all three panels compared to the stable seasonal behavior of the disease. Background colours indicate the four seasons defined by solar solstices and equinoxes: winter (blue), spring (green), summer (yellow), and autumn (orange).


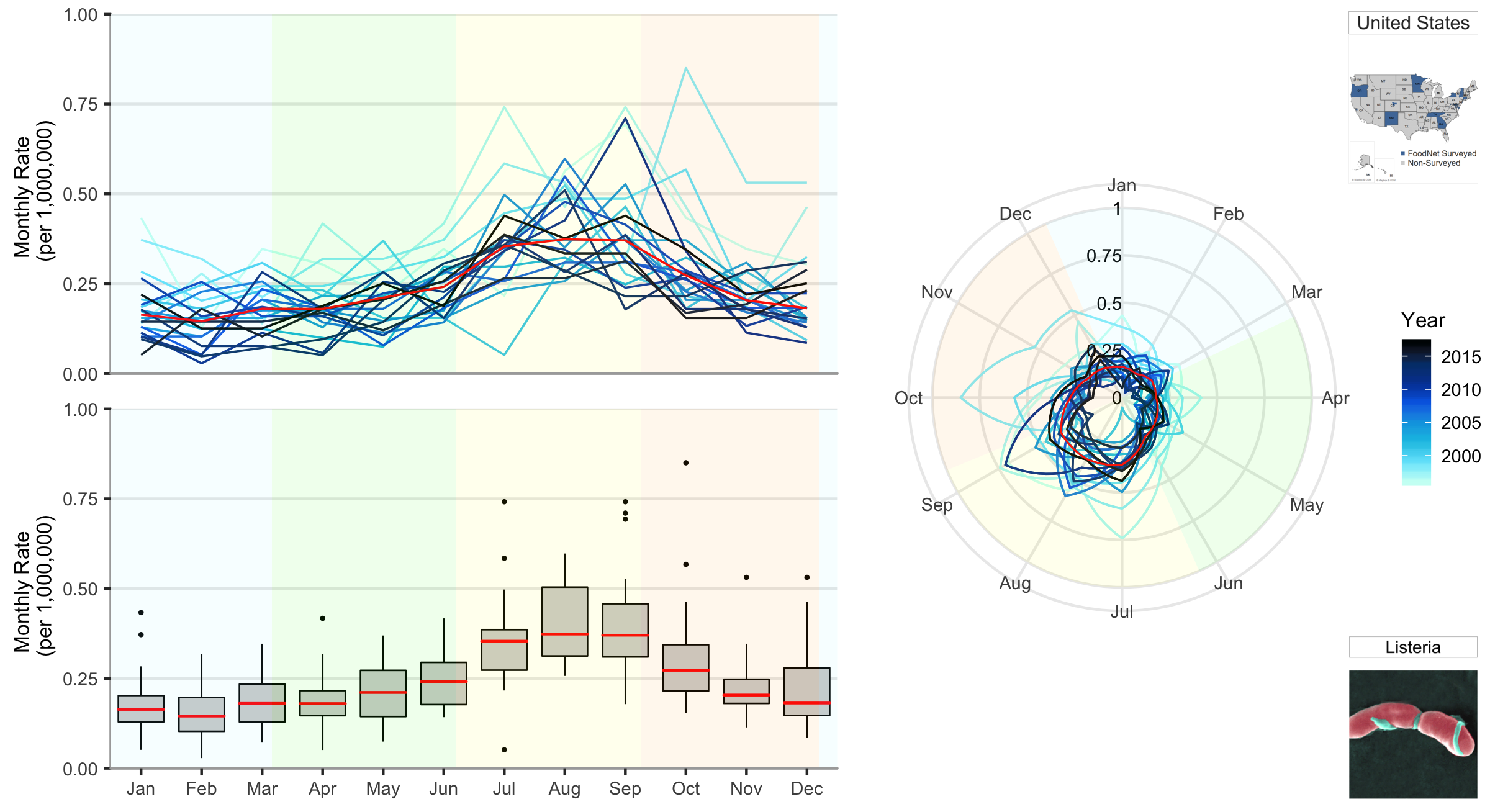


Supplementary Figure S5. A multi-panel plot for visualizing seasonal signatures of listeriosis monthly rates in the US from 1996-2017. While the box plot in the bottom-left panel depicts a stable seasonal signature, the top-left and right panels show the irregular behavior of each annual seasonal signature. Background colours indicate the four seasons defined by solar solstices and equinoxes: winter (blue), spring (green), summer (yellow), and autumn (orange).


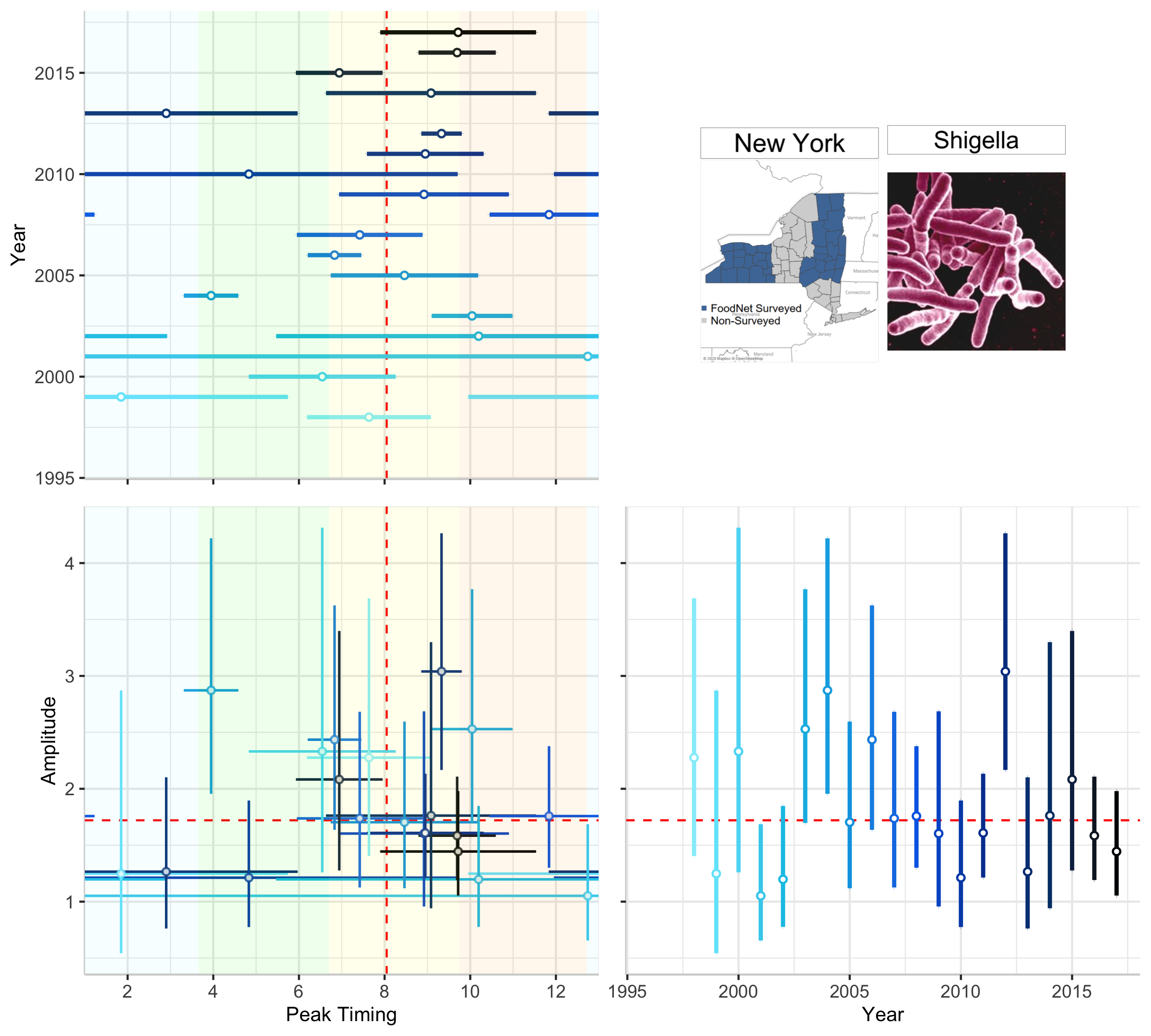


Supplementary Figure S6. A multi-panel plot for visualizing the annual peak timing and amplitude of shigellosis in New York (NY) from 1996-2017. The top-left panel shows the peak timing of shigellosis by year; the bottom-right panel shows the annual amplitude by year. The bottom-left panel shows their combination: a scatterplot between peak timing and amplitude. Background colours indicate the four seasons defined by solar solstices and equinoxes: winter (blue), spring (green), summer (yellow), and autumn (orange). Marker colour intensity indicates more historic vs. more recent data, horizontal and vertical whiskers provide measures of uncertainty, and red lines indicate median peak timing and amplitude across 22 years.


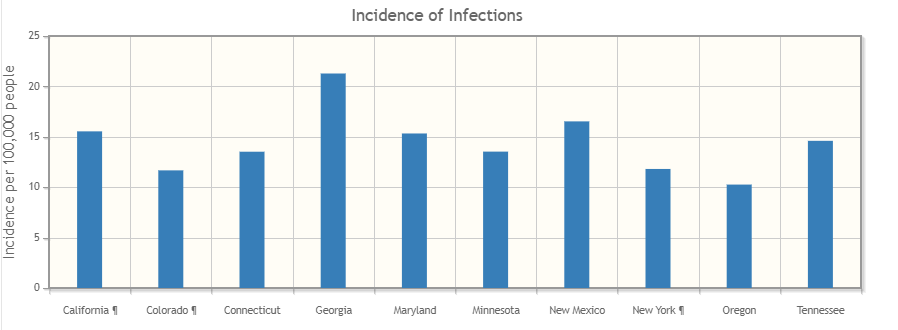


Supplementary Figure S7. A FoodNet Fast bar chart showing the average annual incidence caused by *Salmonella* across all ten FoodNet-surveyed states. This plot was generated on 05 December 2019 using the FoodNet Fast portal [20] by selecting *Salmonella* as the pathogen of interest. The ¶ symbol indicates states where FoodNet does not survey all counties.


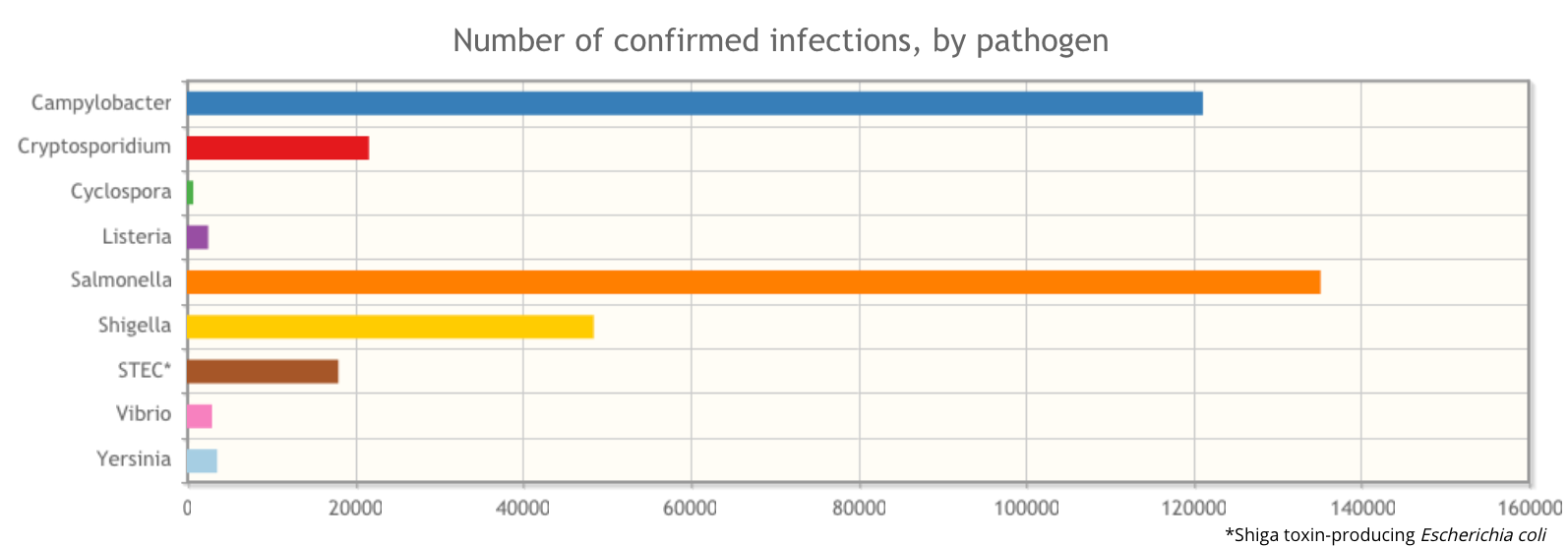


Supplementary Figure S8. A FoodNet Fast bar chart showing the number of confirmed infections by pathogen for the US from 1996-2017. This plot was generated on 05 December 2019 using the FoodNet Fast portal [20] by not selecting any state (default is national estimates).
